# Assessing teachers’ digital competence in primary and secondary education: Applying a new instrument to integrate pedagogical and professional elements for digital education

**DOI:** 10.1007/s10639-023-11848-9

**Published:** 2023-05-05

**Authors:** Katerina Tzafilkou, Maria Perifanou, Anastasios A. Economides

**Affiliations:** 1 SMILE Lab & Department of Economics, University of Macedonia, Egnatia 156, 54636 Thessaloniki, Greece; 2 SMILE Lab, University of Macedonia, University of Macedonia, Egnatia 156, 54636 Thessaloniki, Greece; 3 SMILE Lab, Department of Economics & Information Systems, University of Macedonia, Egnatia 156, 54636 Thessaloniki, Greece

**Keywords:** Digital education, Digital competence, Digital school, Digital skills, Digital teaching, Information literacy, Teachers’ professional development

## Abstract

Teachers’ digital competence (DC) is an important condition for the effective application of technology in education. Although several DC tools have been designed, adjustments to digital education and pedagogical or professional components are still scarce. Therefore, this study aims at developing a new instrument for assessing teachers’ DC regarding their pedagogical and professional activities in the context of digital school and digital education. The study also examines the teachers’ total DC scores and explores the differences between teacher profiles on a sample of 845 teachers in primary and secondary education in Greece. The final instrument comprises 20 items allocated in six components: 1) *Teaching preparation*; 2) *Teaching delivery & students’ support*; 3) *Teaching evaluation & revision*; 4) *Professional development*; 5) *School’s development*; and 6) *Innovating education*. The PLS-SEM analysis indicated the validity and reliability of the model in respect to its factorial structure, internal consistency, convergence validity, and model fitness. The results revealed DC inefficiency among teachers in Greece. Primary school teachers reported significantly lower scores in *Professional development* and *Teaching delivery & students support*. Female teachers reported significantly lower scores in *Innovating education* and *School's development*, but they reported higher scores in *Professional development*. The contribution and practical implications are discussed in the paper.

## Introduction

Digital competence (DC) has become a major issue in education, because not only of the digitalization of the society and economy but also of the emerging digital education landscape. Digital education encompasses several challenges including both students’ and teachers’ attitudes and digital skills. For instance, engaging students online depends both on the students’ digital skills and on the teachers’ ability to efficiently apply digital technologies in digital education and adjust engagement-boosting learning theories (e.g., constructivism and connectivism) to the online context (Bates, 2019). It is well acknowledged that both teachers and students need digital skills to effectively participate in digital education (Hämäläinen et al., 2021). Teachers should be equipped with all the necessary digital skills to efficiently support their students in digital education (e.g., Lucas et al., 2021; Reisoğlu & Çebi, 2020; Winthrop, 2020). However, most recent studies show a lack or inefficiency of teachers’ digital competence (e.g., Fernández-Batanero et al., 2020; Ottenbreit-Leftwich et al., 2018) as well a lack of teachers’ training on digital teaching (e.g., Pérez-Navío et al., 2021; Portillo et al., 2020; Yoon, 2022). Therefore, it is necessary to provide them with up-to-date frameworks to sufficiently assess their digital skills in the current era of digital education.

The teachers’ perceived digital competencies and online readiness are proved to be influenced by personal and contextual factors (Almerich et al., 2016; Lucas et al., 2021). Research findings though show inconsistencies, for instance about the role of gender and age (e.g., Badiozaman et al., 2021; Lucas et al., 2021; Pérez-Calderón et al., 2021; Tondeur et al., 2021; Yoon, 2022). For instance, while women and older in age groups of teachers tend to report lower levels of perceived DC, there are several studies showing no significant differences between gender and age groups. To provide the research community with rich and updated insights on the role of teachers’ personal characteristics, it is crucial to deeper examine them and extend the findings in the context of digital education.

Although several frameworks and instruments have been suggested to assess the teachers’ DC, most of them are based on previously established Information and Communication Technologies (ICT) skills’ dimensions. For example, the DigCompEdu (2021) framework is widely applied to assess the teachers’ DC across different components, focusing though on pedagogical practices and the integration of digital tools in teaching practice. As proved, technological competences tend to affect the teachers’ pedagogical skills and professional use, while pedagogical skills tend to affect the professional dimensions (Suárez-Rodríguez et al., 2018). To the best of our knowledge, there are not any recent frameworks considering the teachers’ ability to apply their digital skills in both their pedagogical and professional activities.

Furthermore, most previous studies focused on the digital competence of pre-service teachers (e.g., Çebi & Reisoğlu, 2022; Gudmundsdottir & Hatlevik, 2018; Kimm et al., 2020; Lázaro-Cantabrana et al., 2019; Yoon, 2022), and not of in-service teachers. Therefore, the current study assesses the digital competences of in-service teachers in primary and secondary education.

This study aims to fill the gaps, by proposing and testing a measurement tool while integrating pedagogical and professional elements related to digital school and distance education. This was accomplished through a voluntary self-administered online survey with 845 teachers at Greek primary and secondary schools. Moreover, the influences of the teacher profiles and personal and contextual factors on digital competence were considered.

Driven by the above-mentioned facts, the related research objectives are as follows:

- RO1: To evaluate a measurement model to examine the teachers’ ability to digitally perform teaching and other professional activities, in the context of digital education, and measure the digital competence of teachers in primary and secondary education in Greece, across the model’s components.
- RO2: To explore differences between teachers’ profiles (according to individual and contextual factors like gender, age, teaching subject, teaching stage, and educational level) on the model’s dimensions.

The findings of the study are expected to bring theoretical and practical implications. First, the results deepen the theoretical understanding on the teachers’ digital skills in the context of digital education and reveal the status quo of the teachers’ DC in primary and secondary education in Greece. Moreover, they present the role of teacher characteristics on DC. A second contribution of the study is to provide policy makers, researchers, and primary and secondary education institutions with valid, comprehensive, and up-to-date measurement models to assess the teachers’ DC towards digital education and professional development. The suggested measurement model is expected to shed light on the teachers’ DC strengths and weaknesses, as well as on the potential necessity for specialized digital technologies and teacher training programs on digital education.

## Theoretical background

### Digital competence

The traditional perspective of DC corresponds to a person’s “ability to use digital technologies in a critical, collaborative, and creative way” (European Commission, 2019; Hatlevik et al., 2015). OECD (2016) also highlights the important role of problem-solving proficiency using digital devices and the frequency of using digital technologies. Educational researchers in DC have mainly examined the students’ and teachers’ perceived levels of digital skills and knowledge across various dimensions. It is supported that a student’s perceived DC reflects their ICT-based knowledge and skills which they can use to do ICT-related tasks (Meng et al., 2019). Whereas a teacher’s perceived DC reflects a more complex concept, co-considering aspects of social, ethical, pedagogical, and attitudinal dimensions (Engen, 2019; Lucas et al., 2021).

Although there are several studies on digital competence in education, there is a misconception on the digital competence of the teachers’ digital competence that includes pedagogical aspects of ICT (Pettersson, 2018). Overall, researchers agree that the definition of what constitutes teachers’ digital competence varies (Eurydice Report, 2019).

### Teachers’ digital competence frameworks

Various national and international organizations developed frameworks to assess the teachers’ digital competence. One popular framework for teachers’ digital competence is the European Framework for the Digital Competence of Educators—DigCompEdu (Redecker, 2017), which is based on the previous version of DigComp framework for citizens (Carretero et al., 2017). DigCompEdu describes 22 competences allocated in the following six dimensions: 1) professional engagement, 2) digital resources, 3) teaching and learning, 4) assessments, 5) empowering learners, and 6) facilitating learners’ digital competence. DigCompEdu addresses educators at all levels of education and focuses on their skills to integrate technology in education, rather than on traditional technical skills. In particular, the DigCompEdu questionnaire items are focused on the pedagogical aspects of preferred communication channels, educational resources, collaborative learning tools, assessment tools and strategies, student course-engagement digital activities, and student’s digital empowerment and encouragement. Several European education systems have developed specific teachers’ digital competence frameworks based on DigCompEdu (Eurydice Report, 2019). The DigCompEdu framework is widely discussed in the study of Caena and Redecker (2019) to show how it reflects the teachers’ updated digital competencies for twenty-first century challenges.

The ‘DigCompEdu Check-In’ tool has been developed as a self-reflection tool for educators and is currently tested in various teacher groups across Europe (DigCompEdu, 2021). The online tool supports different translated versions for different teaching stages such as primary and secondary education, higher education, adult education or continuous professional development, early childhood education and care.

Before DigCompEdu, UNESCO (2018) had developed the ICT Competency Framework for Teachers (ICT CFT) concerning both in-service and pre-service teacher training. The ICT CFT tool consists of 18 competence items allocated in six dimensions of teachers’ professional practice. Many educational systems have adopted and adjusted UNESCO’s ICT CFT to their needs and educational context (Eurydice Report, 2019; UNESCO, 2018).

The International Society of Technology in Education (ISTE, 2018) developed the ISTE Standards for Teachers that provides guidelines for essential technology knowledge and skills. Furthermore, several countries have developed National frameworks combining items from more than one framework. For example, Estonia developed the Standards for Learning, Leading and Teaching in the Digital Age based on DigCompEdu and ISTE (2018), while Ireland developed the Digital Learning Framework which is based on DigCompEdu and UNESCO ICT CTF (Eurydice Report, 2019).

Another popular framework concerning the teachers’ technological knowledge is the Technological Pedagogical Content Knowledge (TPACK), proposed by Mishra and Koehler (2006). The framework describes three basic components of knowledge during teaching and learning, namely technology, content, and pedagogy. TPACK describes the three main components of technological knowledge, pedagogical knowledge, and content knowledge as well as four intersectional components, namely, technological content knowledge, pedagogical content knowledge, technological pedagogical knowledge, and technological pedagogical content knowledge (e.g., Schmid et al., 2020). Several studies have recently applied and (re)validated TPACK to assess the teachers’ digital skills across different teaching stages and domains (e.g., Schmid et al., 2020).

Finally, several researchers used the International Computer Driving License (ICDL, 2018) to assess the teachers’ digital competence, or developed web-based tools to efficiently integrate the framework in educational institutions (e.g., Idrizi et al., 2018). ICDL can be applied in any field of occupation and has been widely adopted by many countries and institutions to evaluate a person’s knowledge on basic ICT concepts like computer basics, word processing and spreadsheets, typing skills, online essentials, online collaboration, image editing, security, presentation, and database.

### Teachers’ digital competence studies

Recently, several empirical studies applied or adjusted previously defined frameworks to assess the teachers’ digital skills in different teaching levels (e.g., pre-service teachers, in-service teachers).

Lucas et al. (2021) validated a DigCompEdu-based instrument on a sample of 1071 teachers from primary and secondary education in Portugal. Their study confirmed previous suggestions on the need to revise and provide better translations to the DigCompEdu items. The findings revealed significant differences across its DC components, based on gender (men reported higher skills than women), and based on age (younger teachers were more competent on using digital technologies than older teachers).

Zhao et al. (2021) applied a 56 items questionnaire on 536 in-service teachers of higher education in China. The instrument was based on the European DigComp framework (Carretero et al., 2017) measuring four components: 1) information and data literacy, 2) communication and collaboration, 3) digital content creation, and 4) safety and problem solving. The results revealed medium to high levels of perceived skills, while male teachers reported higher values than female teachers across all the DigComp components.

Based on the ISTE (2018) Standards for Educators, De León et al. (2021) validated a 72-question test to assess the digital literacy and digital pedagogy of pre-service teachers. A panel of 7 experts evaluate the clarity, quality, and relevance of each subtest question. In a pilot, the test was administered to 88 undergraduate teacher preparation candidates. Finally, the results of the pilot and the expert reviews were combined to demonstrate internal consistency and content validity of the test.

Badiozaman et al. (2021) validated the instrument of Martin et al. (2019) for teachers’ online teaching competence during the pandemic. Their research was conducted on 174 higher education teachers in Malaysia. The applied instrument included 32 items measuring four components of 1) course design, 2) communication, 3) time management, and 4) technical competency. The respondents rated high perceived skills of online teaching particularly in the components of course design and communication. No gender or other individual differences were observed in the examined constructs.

Pérez-Calderón et al. (2021), applied and validated the DigCompEdu framework on 109 teachers in Spain. The results revealed high levels of competence in most components. A deficit was observed in the use of digital tools for Augmented Reality learning material since it is a quite novel resource. The gender-based analysis revealed significantly lower scores of perceived DC in women than men.

Schmid et al. (2020) applied and validated the TPACK framework on 117 upper secondary school teachers, while attending a teacher training program in Switzerland. The authors came up with a short 28-items questionnaire to assess the teachers’ TPACK-related skills.

Redmond et al. (2021) designed a mixed-method study to evaluate Australian primary teachers’ self-assessment of their digital technology, based on the standards of the Australian Curriculum Assessment and Reporting Authority (ACARA, 2019). The quantitative part of the survey followed the TPACK framework Their findings revealed high average scores of teachers’ confidence in digital technologies activities, and teachers in the foundation year reported the highest scores.

Falloon (2021) designed and suggested a comprehensive framework extending TPACK, to integrate some broader competences of discipline and content knowledge, as well as personal-ethical and personal-professional dimensions. The conceptual model of the new framework is deeply analyzed, but there is not yet any statistical validation of the model on actual teacher population.

Ghomi and Redecker (2019) validated the DigCompEdu Check-In instrument on 355 German teachers. They found that teachers with more years of experience in using technologies in teaching indicate higher scores of digital competences. The authors detected some differences in digital competence between different teaching subjects, between computer science and non-computer science teachers.

Based on the DigCompEdu, Perifanou et al. (2019) designed a questionnaire to evaluate the teachers’ confidence in applying digital resources in their teaching practices. Their sample consisted of 300 teachers from Primary and Secondary Education from four European countries (Cyprus, Greece, Slovakia, Spain). They found that there are significant differences among the four countries regarding the teachers’ confidence. In addition, teachers’ educational qualification and continuous professional development level were identified as predictors of the teachers’ confidence.

Earlier, Benali et al. (2018) had explored the DigCompEdu Check-In instrument finding a good distribution of competence levels across 160 Moroccan English teachers. Teachers with a higher digital teaching confidence or more years of teaching experience reported higher scores in the digital competence components. The results also revealed low levels of competence in self-regulated learning, digital assessment, accessibility and inclusion and personalization.

Cattaneo et al. (2022) validated DigCompEdu framework and assessed the level of digital competence among 597 vocational teachers in Switzerland. The results of the study showed that the overall level of digital competence among vocational teachers was relatively high. However, there were significant differences in the level of competence across the six areas of the DigCompEdu framework. The authors found that teachers had the highest levels of competence in the areas of "Digital Resources" and "Teaching and Learning", while they had the lowest levels of competence in the areas of "Data Protection and Digital Security" and "Communication and Collaboration". The study also revealed several factors that contribute to the development of digital competence including personal characteristics, such as age and gender, as well as institutional factors, such as access to resources and support from colleagues and management.

A lot of research on DC has also been conducted on pre-service (students) teachers (e.g., Çebi & Reisoğlu, 2022; Gudmundsdottir & Hatlevik, 2018; Kimm et al., 2020; Lázaro-Cantabrana et al., 2019; Yoon, 2022) where researchers also applied previously established frameworks, like the DigComp (e.g., Barnard et al., 2020). Evidence suggests that young pre-service teachers need further training on the DigComp components of communication and collaboration, information and data literacy, digital content creation, safety, and problem solving (Reisoğlu, & Çebi, 2020). Like in-service teachers, personal characteristics are proven to affect pre-service teachers’ attitude and DC as well (Tondeur et al., 2021).

As observed, previous studies ignore the need for teachers to efficiently accomplish other professional activities (such as school management, education innovation, etc.) besides teaching. In addition, there are few studies or even conflicting results regarding the effect of teachers’ personal factors and contextual factors to teachers’ digital competence. Therefore, the current study aims at considering a wide spectrum of teachers’ digital competences for teaching, professional self-development, school’s development, and innovating education as well examining the effect of personal (e.g., gender, educational level) and contextual factors (e.g., teaching subject, school level) to the digital competence of in-service teachers in primary and secondary education in Greece.

## Methodology

### Instrument

**Fig. 1:**
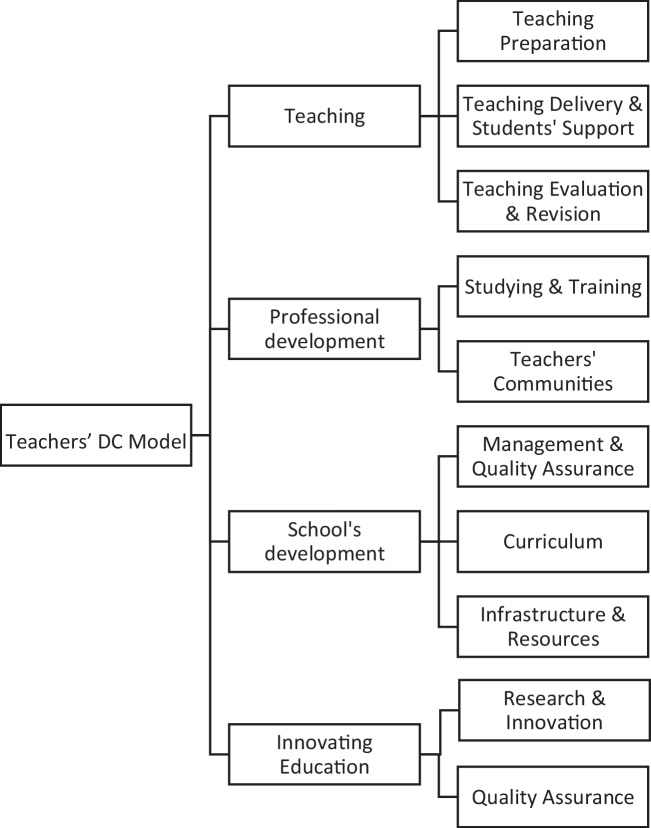
Model’s conceptual framework

A 20-item questionnaire was considered reflecting the teachers’ digital skills towards integrating digital tools and technologies into the teaching practice (e.g., video conferencing platforms, learning management systems, digital assessment tools, etc.) and professional development (e.g., management and scheduling of classes, participation in online teaching communities, management of the school’s infrastructure, development of innovating educational resources, etc.) The initial items were retrieved from the study by Perifanou et al. (2021) who assessed the teachers’ digital skills readiness to cope with digital education, emerged due to Covid-19. The items in Perifanou et al. (2021) were conceptualized into teachers’ pedagogical and professional activities reflecting four core dimensions: 1) *Teaching,* 2) *Professional development;* 3) *School’s development;* and 4) *Innovating education*, as depicted in Fig. 1. The cited model has not been statistically validated in previous studies.

The questionnaire was developed in English and then translated into Greek, the native language of the teachers. The translation was made by three experts in the field of Technology Enhanced Learning (TEL) to ensure linguistic equivalence. All items were measured on a 5-point Likert scale (1: not at all to 5: excellent) responding to the question “To what extent (intensity, quantity, and frequency) do you use digital tools for the following?”. The items of the instrument are based on the questionnaire developed in Perifanou et al. (2021) and are depicted in Table 6 in Appendix.

### Sample characteristics and data collection

A population of 5800 primary and secondary education teachers was invited to participate in the survey. A non-probability sampling approach was followed, since the sampling frame was available to primary and secondary teachers who were enrolled in the National Project of “Advanced Training for the Utilization and Application of ICT in the teaching practice”, in Greece. The teachers were invited in the survey through emails, after the completeness of the program. Finally, 845 teachers (631 female, 214 males; 614 secondary education, 231 primary education; average age: 41–50) successfully completed the survey. 50% of the participants had a master’s degree, 45% had a bachelor’s degree, and 5% had a doctorate degree or higher. Many of the participants (n = 233) were teaching in primary education, 146 were foreign language teachers, 108 were teachers of physical education, 79 were teaching computer science, 59 were teaching literature, 55 were teaching finance or business administration, 48 were mechanic engineers, while the rest of them were teaching fine arts, special education, or other.

All participants approved their volunteer participation, and data collection and manipulation were applied according to the principles of the institute’s ethical committee.

### Data analysis

Overall, the data analysis included a PLS-SEM analysis to assess the validity of the DC scale on our sample of Greek teachers in primary and secondary education, and descriptive statistics to present the status quo of teachers’ digital competence, as well as analysis of variance of the VET teachers’ profiles to investigate possible differences in digital competence across profiles. In the following paragraphs, we provide additional details on each analytical step.

The validation of the model was achieved through a PLS-SEM approach because this study explores and extends previous theories (Hair et al., 2017a, b). Researchers in the field (Bentler & Bonett, 1980; Dijkstra & Henseler, 2015) showed that PLS-SEM can consistently mimic common CB-SEM approaches and is adequate to measure and validate the structure of a model. Previous studies have also chosen PLS-SEM to validate their models (Tzafilkou et al., 2021) since it is considered appropriate for complex models and social science research (Asyraf & Afthanorhan, 2013). For these reasons, this study applied a PLS-SEM methodology to measure and validate the proposed teachers’ digital skills instrument. The validation was executed through the SmartPLS software.

The descriptive statistics were calculated through the SPSS software. Finally, to examine any significant differences among teacher groups of different gender and teaching subjects, non-parametric statistical methods (Mann Whitney and Kruskal Wallis) were conducted due to the non-normal distribution in the data. The normality criteria were based on the Shapiro–Wilk test (1965), and all variables in the dataset revealed non normal distributions (p < 0.05).

## Results

### Validity of the teachers’ digital competence scale

As described in the sections below, the factor analysis and PLS-SEM results generated a valid DC model composed of 20 items and six main components as follows: 1) *Teaching Preparation (TP)*; 2) *Teaching Delivery & students’ support (TD)*; 3) *Teaching Evaluation & Revision (TER)*; 4) *Professional Development (PD)*; 5) *School’s Development (SCD)*; and 6) *Innovating Education (IE)*. Overall, the teaching components formed three distinct constructs, while the rest subcomponents were merged reflecting their parent component.

#### Model fitness

The value of Root Mean Square Error of Approximation (RMSEA = 0.076) in this sample indicates an acceptable fit according to the defined acceptance criteria (Bandalos, 2018; Henseler et al., 2014). RMSEA is used in PLS-SEM analysis to avoid model misspecification. Overall, a value less than 0.10 or of 0.08 is considered a good fit. The rest of the model fit indices were Chi-Square = 2581.652 and Normed Fit Index (NFI) = 0.754.

#### Confirmatory factor analysis

As depicted in Table 1, all criteria for internal consistency and convergence validity are satisfied: all values of Cronbach alpha demonstrate internal consistency exceeding 0.6 (Dijkstra & Henseler, 2015), the values of composite reliability exceed 0.6, and average variance (AVE) values range from 0.6 to 0.785 (AVE > 0.5) (Bandalos, 2018; Muthén & Muthén, 2012). Finally, as depicted in Table 2 the scores of the loading factors are above the minimum threshold of 0.5 (Awang, 2012).

**Table 1:** Descriptive statistics and results for construct reliability and convergent validity for the measurement model (acceptable threshold values in brackets) *N* = 845

Component	Mean [1,5] (SD)	Cronbach's Alpha (> 0.6) *	Composite Reliability (> 0.6) *	Average Variance Extracted (AVE) (> 0.5) *
TP: Teaching preparation	2.845 (0.891)	0.675	0.816	0.600
TD: Teaching delivery & students’ support	2.457 (0.929)	0.848	0.891	0.621
TER: Teaching evaluation & revision	2.349 (1.006)	0.852	0.900	0.693
PD: Professional development	3.282 (1.113)	0.730	0.881	0.787
SCD: School's development	2.198 (0.937)	0.832	0.889	0.667
IE: Innovating Education	1.918 (1.006)	0.726	0.879	0.785

* Bandalos (2018), Muthén and Muthén (2012)

**Table 2:** Outer factor loadings

	Teaching preparation	Teaching delivery & students’ support	Teaching evaluation & revision	Professional development	School’s development	Innovating education
TP1	0.794					
TP2	0.656					
TP3	0.860					
TD1		0.790				
TD2		0.774				
TD3		0.734				
TD4		0.813				
TD5		0.826				
TER1			0.781			
TER2			0.824			
TER3			0.866			
TER4			0.858			
PD1				0.875		
PD2				0.899		
SCD1					0.710	
SCD2					0.851	
SCD3					0.831	
SCD4					0.866	
IE1						0.875
IE2						0.897

#### Discriminant validity

The cross loadings of the items for every factor can be viewed at Table 7 in Appendix A. To further ensure the validity of the construct, the discriminant validity was evaluated using the criterion of Fornell and Larcker (1981), which is the most widely used method. According to this criterion the square root of each construct’s AVE should have a greater value than the correlations with other latent constructs. Table 3 shows that the instrument supports the discriminant validity between the three constructs (Fornell & Larcker, 1981).

**Table 3:** Discriminant validity for the measurement model (values in bold: the square root of the average variance extracted for each construct)

Component	TP	TD	TER	PD	SCD	IE
TP	0.775					
TD	0.699	0.788				
TER	0.644	0.816	0.833			
PD	0.470	0.556	0.476	0.887		
SCD	0.709	0.435	0.715	0.435	0.817	
IE	0.613	0.727	0.711	0.441	0.813	0.886

*TP* Teaching preparation, *TD* Teaching delivery & students’ support, *TER* Teaching evaluation & revision, *PD* Professional development, SCD: School's development, *IE* Innovating education

### Descriptive statistics

The general descriptive statistics of all variables are presented in Table 4. As depicted, teachers’ DC are at medium levels across most DC components. Innovating education suffers the most since respondents scored the lowest mean values (mean = 1.91/5.00) while Professional development received the highest scores of perceived DCs (mean = 3.28/5.00).

**Table 4:** Descriptive statistics of the study variables and the digital competency total score (N = 845)

	Mean [1, 5]	Std. Deviation
TP	2.8450	0.8914
TD	2.4573	0.9297
TER	2.3491	1.0062
PD	3.2000	1.2910
SCD	2.1981	0.9378
IE	1.9189	1.0063
DC total score	2.4947	0.8231

The DC total score is calculated as the mean value of the scale components

### Digital competence differences between teacher profiles

Table 5 depicts the statistical results of the comparisons in differences between teachers’ profiles.

**Table 5:** Significant differences in DC model components among teacher profiles (Mann–Whitney and Kruskal–Wallis test)

	TP	TD	TER	PD	SCD	IE
Grouping variable: gender						
Mann–Whitney U	61,630.000	64,346.000	63,522.000	57,267.000	54,573.500	58,396.500
Wilcoxon W	261,026.000	263,742.000	262,918.000	80,272.000	253,969.500	257,792.500
Z	-1.920	-1.030	-1.299	-3.350	-4.212	-3.052
Asymp. Sig. (2-tailed)	0.055	0.303	0.194	0.001*	0.000*	0.002*
Grouping variable: teaching subject						
Mann–Whitney U	35.755	41.804	24.418	41.256	42.584	28.741
Wilcoxon W	11	11	11	11	11	11
Z	0.000*	0.000*	0.011*	0.000*	0.000*	0.002*
Grouping variable: teaching stage (primary/secondary)						
Mann–Whitney U	68,874.500	62,853.000	66,957.000	58,442.500	66,751.000	69,776.000
Wilcoxon W	95,670.500	89,649.000	93,753.000	85,238.500	93,547.000	96,572.000
Z	-0.650	-2.556	-1.256	-3.978	-1.323	-0.373
Asymp. Sig. (2-tailed)	0.516	0.011*	0.209	0.000*	0.186	0.709
Grouping variable: educational level						
Chi-Square	37.149	36.724	34.372	69.912	36.873	43.842
df	2	2	2	2	2	2
Asymp. Sig	0.000*	0.000*	0.000*	0.000*	0.000*	0.000*

*. Statistical significance at level p = 0.05

## Discussion

### Principal findings

#### Model validity

The study results demonstrate that suggested model is valid and reliable in respect to its factorial structure, internal consistency, convergence validity, and model fitness.

#### Teachers’ DC skills

The descriptive statistics in Table 2 results indicate that teachers’ DC are at medium levels across most components. Overall, the highest mean score was observed for Professional Development (PD) and the lowest for the Innovative Education (IE) competence. The mean of the total digital competence score was at a medium level (2.5/5.0). The findings align with previous studies reporting that teachers had insufficient online teaching skills (OECD, 2020) and they lacked the appropriate digital skills for teaching, guiding, and assessing their students (e.g., Ottenbreit-Leftwich et al., 2018; School Education Gateway, 2020).

However, compared to some studies in other countries (e.g., Cattaneo et al., 2022 in Switzerland), Greek teachers seem to report lower levels of DC, reflecting the need to reinforce the teachers’ digital competencies in Greece.

#### Differences according to gender

Regarding gender differences in digital skills, literature suggests that men have higher digital skills than women in most countries (ITU, 2019; Perifanou & Economides, 2020). The results in Table 5 show that male teachers reported significantly higher scores than female teachers in the SCD and IE components, while female teachers reported higher values of perceived Professional development (PD). This result extends previous findings by indicating that men and women show differences in certain DC components. For instance, Pérez-Navío, et al. (2021) found that men perceived higher competencies in information processing, while women reported higher competency in information searching for academic activities. Lucas et al. (2021) found that men claimed higher digital technology-related competence but lower digital content knowledge and teaching methods than women. However, Almerich et al. (2016) found that female teachers hold higher pedagogical competencies than male teachers. Other studies did not find any significant gender difference in the teachers’ perceived digital skills (Prieto et al., 2020). Similar research on students revealed gender differences, where male students expressed higher levels of perceived computer and digital skills than female students (Grande-de-Prado et al., 2020). Overall, the contradicting results imply that gender-based differences should be studied in detail focusing on the distinct digital skills’ components and considering factors that might affect the results or research limitations. For instance, all the discussed instruments assess perceived skills and do not apply any practical assessment or direct observation methodologies. The fact that women generally report lower levels of ICT self-efficacy can possibly explain the current findings on existing gender gaps in some DC components (SCD, IE). Another possible explanation might be the negative emotional effects of the pandemic to the females’ teachers’ population compared to men (Portillo et al., 2020).

#### Differences according to teaching subject

Interestingly, as depicted in Table 6 in Appendix A, teaching subjects indicated significant differences across all the pedagogical and professional components. Teachers of Informatics reported the highest scores in all components, while the lowest scores were rated by Literature, Foreign Language, and Primary Education teachers. This finding aligns with previous studies indicating significant DC differences between computer science and non-computer science teachers (Ghomi & Redecker, 2019). As regards to Professional development, the lowest values were reported by primary education teachers, while the highest by Special education, Fine Arts, and Informatics teachers. These findings reflect the important role of ICT knowledge and technical background or experience on the teachers’ digital skills and efficient online teaching. Also, by analyzing the two groups of primary and secondary education teachers, significant differences were observed in the components of PD and TD where teachers in primary education reported significantly lower values. Overall, the above findings strongly support the need for ICT oriented teacher training programs.

#### Differences according to educational level

Regarding the teachers’ educational level, we observed that teachers holding a doctorate degree reported the highest values in all components, while those holding only a bachelor’s degree reported the lowest ones. A further statistical analysis (a 2 independent sample Mann Whitney Test) between master and doctorate degree teachers revealed that there are significant differences between these two levels as well, but only in two components: SCD and ED. This finding reflects the large difference between teachers of bachelor’s degree and those of master or above (doctorate), implying that continuing education can positively affect the teachers’ level of digital skills. Again, this finding supports the urgent need for the design and development of more teacher ICT training programmes.

Finally, no significant differences were reported according to age, aligning with previous findings (Benali et al., 2018; Tondeur et al., 2021). However, other studies have reported that young teachers have higher digital competence (Castéra et al., 2020; Hämäläinen et al., 2021), hence more research is suggested.

### Contribution and practical implications

Overall, the study provides valuable insights into the level of digital competence among teachers and the factors that influence its development. The results also highlight the importance of ongoing professional development and support for teachers in primary and secondary education, to ensure that they have the necessary digital competence to effectively teach in a digital age. Based on the results on the teachers total DC scores, this study proposes that further actions and teacher training programmes should be organized to enhance the teachers’ DC in Greece, confirming recent suggestions that teachers need appropriate training (e.g., Benali et al., 2018; Cabero-Almenara et al., 2021; Fernández-Batanero et al., 2020; Gudmundsdottir & Hatlevik, 2018; Tondeur et al., 2021; Yoon, 2022).

Additionally, the suggested scale provides a comprehensive framework that can be applied alone or in combination with other tools to evaluate teachers’ DC across pedagogical, institutional, and professional development practices in the context of DE. The application of the suggested DC model by teachers, policy makers, and institutions can be useful in several dimensions:

- **Teacher training programs**: The teaching professions face emergency changing demands due to the explosion of online teaching and ubiquitous technologies (DigCompEdu, 2021). EU teachers report that ‘ICT skills for teaching’ is one of their greatest training needs (OEDC, 2019). For this, applying up-to-date competence frameworks will contribute to the identification of DC weaknesses and the need for specialized ICT teacher training programs. The suggested DC model is appropriate to leverage the skills and weaknesses in both professional and pedagogical teaching practices, providing a holistic and clear image of the teachers’ DC training needs in the context of digital education.
- **Teacher DC development:** By individually applying the model, teachers can identify their strengths and weaknesses in both their professional and pedagogical ICT practices. This will assist them in efficiently designing their learning paths to develop their digital competence. The tool can be used in adjustment or in combination with the DigCompEdu tool as well to assess broader aspects of teachers’ professional activities and remote teaching.

### Limitations and future research

One possible limitation of this study regards generalization since the study was conducted in Greece and on a particular teachers’ population who participated in a national ICT teacher training program. Different teacher populations (e.g., another country, university faculty) would possibly yield differentiated outcomes. Also, teachers voluntarily participated in the study. So, the perceptions of teachers who did not want to answer the questionnaire were not taken into consideration.

Another limitation is related to the self-assessment nature of the instrument. Although self-reporting is an easy and fast process, it depends on the teachers’ perceptions about their digital competence. However, their perceptions may depend on their personality, self-awareness, emotions, etc. Thus, they may overestimate or underestimate their digital competence. Future research could objectively assess the teachers’ digital competence by asking them to solve specific problems and exercises or answer exam-style questions.

Finally, the results of the gender-based analysis might be biased due to the underrepresentation of men participants in the study. Also, other factors might have affected the gender-based results like for instance the fact that there are more male teachers of Informatics than female teachers, while there are more female teachers of Literature or Foreign Languages than male teachers. So, further research is needed to examine whether gender plays an actual role in teachers’ digital skills, independently from the teaching subject.

## Conclusions

This study measures and validates the suggested DC scale to assess teachers’ digital competence. The scale takes into consideration both professional and pedagogical aspects attempting to extend previous DC frameworks in the context of digital schools and digital education. The suggested scale is composed of 20 items and six components: 1) Teaching preparation; 2) Teaching delivery and students’ support; 3) Teaching evaluation & revision; 4) Professional development; 5) School’s development; and 6) Innovating education. The statistical results demonstrated the model’s validity and reliability, showing internal consistency, convergence validity, and accepted model fit criteria.

The descriptive statistics results revealed unsatisfying levels of DC among Greek teachers in primary and secondary education, confirming previous studies that reported insufficient DC among teachers. The examination of personal and contextual attributes of gender, teaching subject, and educational level revealed significant differences across the DC constructs.

The results of this study are useful for policy makers and educators to identify DC strengths and gaps and design tools and strategies to achieve efficient digital teaching and further professional development.

## Data Availability

The data that support the findings of this study are available from the corresponding author upon reasonable request.

## Appendix A

**Table 6:** Instrument items and initial components

Component	Sub-component	Item	
Teaching	Teaching preparation: TP	TP1	I use digital tools to identify and analyze the needs and characteristics of students, as well as the available resources, devices, and infrastructure for my lessons
		TP2	I use search engines, digital repositories, databases, and test banks to explore, find, and evaluate existing educational resources (e.g., OERs) using various criteria, metadata filters, and recommender systems
		TP3	I use a digital calendar, time-planning, and project management software to plan, organize, and schedule the most appropriate educational resources for teaching
	Teaching delivery & Students’ support: TD	TD1	I use software for project, school, and classroom management, learning management systems, plagiarism, and security software to manage the lesson, classroom, activities, students, educational resources, devices, and infrastructure, etc
		TD2	I use digital communication and collaboration technologies to interact, communicate and collaborate with students
		TD3	I use digital assessment technologies (e.g., digital quizzes, tests, questions, exercises, assignments, inquiries, web quests) to periodically assess the students’ progress
		TD4	I use digital monitoring tools (e.g., remote desktop control & screen sharing), dashboards, and learning analytics to monitor students’ activities, interactions, relationships, mood, and performance, as well as the teaching process, educational resources, equipment, and infrastructure
		TD5	I use learning management systems, software for reminding, annotations, auto-correction, chatbots, avatars, recommendation systems to guide, feedback, advice, support, and inspire students
	Teaching evaluation & Revision: TER	TER1	I use digital diaries, blogs, concept mapping, mind mapping, digital tools for recording thoughts, voice, photos, videos, self-assessment, etc. to record my thoughts, feelings, behavior, and performance as well as those of my students, as well as the use and quality of educational resources, equipment, and infrastructure, and then to reflect on (and review) them
		TER2	I use information from digital monitoring, evaluation, and reflection tools to continuously adapt my teaching using digital technologies
		TER3	I use digital evaluation tools (e.g., student examination, school management tools, safety, and technology performance evaluation software) to evaluate students, my teaching, educational resources, devices, and infrastructure
		TER4	I use information from monitoring, testing, and evaluation tools to revise the preparation, delivery, and evaluation of my teaching using digital technologies
Professional development: PD	Studying & Training	PD1	Ι use, attend, and study online courses (e.g., MOOCs), OERs, e-books, e-journals, and other digital educational materials for professional development
	Teachers’ communities	PD2	I collaborate and participate in online teacher communities, online conferences & seminars, and other social media for professional development
School's development: SCD	School’s management & Quality assurance	SCD1	I use and manage digital tools for the management & administration of the school (e.g., digital recording attendance and grades, time-planning of classes and events, online collaboration with parents, teachers, other schools, and ministry)
	Curriculum	SCD2	I use and manage digital tools for curriculum development & management
	School’s infrastructure & Resources	SCD3	I use and manage digital tools for the development & management of the school’s infrastructure and resources
	School’s Management & Quality Assurance	SCD4	I use and manage digital tools for the development & management of the school’s quality assurance
Innovating Education: IE	Educational research & Innovation	IE1	I use digital technologies to generate research and innovation in education (e.g., statistical analysis of questionnaires to identify important parameters that affect learning; creation of educational software for the development of simulations)
	Quality assurance in Education	IE2	I use digital technologies to support quality assurance in education (e.g., statistical analysis of student performance & career; statistical analysis of teacher training programs from different schools, regions, and states)

**Table 7:** Cross loadings

	Professional development PD	Innovating education IE	School's development SCD	Teaching delivery & students’ support TD	Teaching preparation TP	Teaching evaluation & revision TER
PD1	0.875	0.376	0.385	0.467	0.427	0.394
PD2	0.899	0.406	0.387	0.517	0.409	0.448
ED1	0.446	0.875	0.674	0.614	0.545	0.602
ED2	0.340	0.897	0.762	0.672	0.541	0.656
SCD1	0.386	0.471	0.711	0.536	0.558	0.453
SCD2	0.355	0.698	0.851	0.619	0.622	0.601
SCD3	0.362	0.689	0.831	0.624	0.572	0.611
SCD4	0.331	0.767	0.866	0.695	0.570	0.653
TD1	0.406	0.656	0.707	0.790	0.633	0.655
TD2	0.535	0.470	0.511	0.774	0.508	0.582
TD3	0.458	0.397	0.414	0.734	0.472	0.578
TD4	0.405	0.662	0.679	0.813	0.543	0.677
TD5	0.413	0.634	0.640	0.826	0.584	0.708
TP1	0.294	0.449	0.544	0.496	0.794	0.456
TP2	0.391	0.304	0.338	0.394	0.656	0.344
TP3	0.417	0.607	0.691	0.679	0.860	0.637
TER1	0.415	0.581	0.567	0.665	0.535	0.781
TER2	0.416	0.526	0.504	0.633	0.499	0.824
TER3	0.362	0.674	0.695	0.723	0.573	0.866
TER4	0.397	0.579	0.603	0.692	0.535	0.858
